# Up-regulation of CacyBP/SIP during rat breast cancer development

**DOI:** 10.1007/s12282-012-0399-1

**Published:** 2012-08-28

**Authors:** Ewa Kilańczyk, Krzysztof Gwoździński, Ewa Wilczek, Anna Filipek

**Affiliations:** 1 Nencki Institute of Experimental Biology, 3 Pasteur Street, 02-093 Warsaw, Poland; 2 Department of Molecular Biophysics, University of Lodz, Lodz, Poland; 3 Department of Pathology, Medical University of Warsaw, Warsaw, Poland

**Keywords:** Breast cancer, CacyBP/SIP, DMBA-induced breast cancer model, β-Catenin

## Abstract

**Background:**

CacyBP/SIP (calcyclin binding protein/Siah-1 interacting protein) was originally discovered in Ehrlich ascities tumor cells but was later found also in many different tumors.

**Methods:**

To better understand the function of CacyBP/SIP in carcinogenesis, we used immunohistochemistry, Western blotting, and RT-PCR assays to study the distribution and level of CacyBP/SIP in mammary tissues after tumor induction in rat with DMBA [(dimethylbenz[*a*]anthracene)]. Application of such a model allowed us to monitor changes in CacyBP/SIP level during development of breast cancer.

**Results:**

We found that both the protein and mRNA levels of CacyBP/SIP gradually increased in pathologically changed tissues and were highest in tumors taken 8 weeks after DMBA treatment. Similar changes as for CacyBP/SIP were detected in the level of β-catenin.

**Conclusion:**

Increase in CacyBP/SIP expression during development of breast cancer, observed early in the mammary tissues with only minimal pathological changes, might suggest an important role of this protein in the process of carcinogenesis.

## Introduction

CacyBP/SIP (calcyclin binding protein/Siah-1 interacting protein) was discovered in Ehrlich ascities tumor cells [1]. Recent studies have indicated that CacyBP/SIP participates in many cellular processes [2] such as regulation of the ERK1/2 pathway [3, 4], organization of the tubulin-actin cytoskeleton [5, 6], differentiation of neuroblastoma cells and cardiomyocytes [5, 7], and regulation of apoptosis [8]. In addition, CacyBP/SIP also seems to play a key role in the development of different cancers. Among them is pancreatic cancer [9–11], in which CacyBP/SIP expression is associated with an aggressive phenotype. Increased CacyBP/SIP expression is also observed in renal cell carcinoma in which the up-regulation seems to be associated with suppression of cell growth and carcinogenesis [12]. Recently, it was reported that CacyBP/SIP could play an important role in colon cancer [13, 14]. Although these data suggest a possible involvement of CacyBP/SIP in carcinogenesis its precise role in this process still remains unclear.

As mentioned above, CacyBP/SIP was discovered in Ehrlich ascities tumor [1], which is of a breast cancer origin. Up to now, only two papers have been published regarding the search for the role of CacyBP/SIP in breast cancer. One of them shows that expression of CacyBP/SIP is significantly decreased in breast cancer tissues [15], whereas the other demonstrates that CacyBP/SIP expression is increased in this tumor [16]. To better understand the role of CacyBP/SIP in breast cancer, particularly during its development, we used a rat model of this tumor. Such a model allowed us to collect and examine tissues at different time points, i.e., at different stages of cancer development, after induction of tumor with a synthetic carcinogen—dimethylbenz[*a*]anthracene (DMBA) [17, 18]. In this work we analyzed the level of the CacyBP/SIP protein and its mRNA in rat mammary cancer samples using immunohistochemistry, Western blotting, and RT-PCR. We found that the level of CacyBP/SIP increases during development of cancer in a manner similar to the level of an oncogene, β-catenin.

## Materials and methods

### Rat breast cancer model

The studies on adult Sprague–Dawley female rats were conducted with approval of the Ethics Committee of the Medical University of Lodz, Poland. Animals were housed in an environmentally controlled room (temperature 23 °C ± 1 °C, 12 h light/12 h dark cycle). Before tissue samples were taken rats were anesthetized and killed by cervical dislocation.

For the studies, three control rats and six rats with DMBA-induced mammary cancer were used, i.e., one control and two DMBA-treated rats for each time point. Rats at 48–50 days of age were gavaged with PBS (control) or 20 mg/kg of DMBA suspended in 1.6 ml of olive oil. Animals were inspected weekly for mammary tumors by palpation. No tumors were detectable 3 and 6 weeks after treatment; thus, two mammary tissue samples were taken from each of the DMBA-treated rats as well as from control animals. Eight weeks after treatment tumors were easily discernible and seven tumors were excised altogether from the two DMBA-treated rats and two mammary tissue samples from the control animal.

### Preparation of tissue extracts, SDS-PAGE, and Western blotting

Control tissues or tumors were homogenized with Polytron, four times for 30 s at 6,000 rpm, in lysis buffer containing 50 mM Tris–HCl pH 7.5, 150 mM NaCl, 1 mM EDTA, 2 mM DTT, and protease inhibitors. Protein concentration was quantified according to Bradford’s procedure and 40 μg of protein was separated by SDS-PAGE (10 %) by the method of Laemmli [19]. Then, the proteins were transferred onto nitrocellulose and identified using appropriate primary antibodies: rabbit anti-CacyBP/SIP affinity purified polyclonal antibody (1:100, made in house), monoclonal anti-β-catenin (1:500, Sigma), or monoclonal anti-GAPDH antibody (1:3,000, Alexis Biochemicals). Blots were washed with TBS-T buffer (50 mM Tris pH 7.5, 200 mM NaCl, 0.05 % Tween 20) and then allowed to react with secondary antibodies, either goat anti-mouse IgG (1:10,000, Jackson Immunoresearch Laboratories) or goat anti-rabbit IgG (1:5,000, MP Biomedicals) conjugated to horseradish peroxidase. After three washes with the TBS-T buffer and two washes with the TBS buffer (50 mM Tris pH 7.5, 200 mM NaCl) blots were developed with the ECL chemiluminescence kit (Amersham Biosciences) followed by exposition against a RETINA X-ray film.

### Immunohistochemistry and immunofluorescence staining

The 4 μm formalin fixed paraffin embedded sections were deparaffinized in xylene and alcohols. Then antigen retrieval was performed by cooking sections in 0.01 M citric buffer, pH 6.0 in a microwave oven. The endogenous peroxidase activity was blocked by incubation with 3 % hydrogen peroxide for 30 min. After this step sections were placed in 2.5 % bovine serum albumin (BSA, DakoCytomation, Germany) to reduce unspecific binding. The affinity purified rabbit anti-CacyBP/SIP (made in house) or anti-β-catenin (BD Biosciences) antibodies were applied at 1:50 dilution in 2.5 % BSA and the sections were incubated overnight at 4 °C. Antigen detection was performed using the anti-rabbit Envision + System-HRP Labelled Polymer (DakoCytomation, Germany) and visualization was carried out using 3,3′-diaminobenzidine (DAB) (DakoCytomation, Germany) as a chromogen. After this immunohistochemical procedure sections were counterstained with hematoxylin to visualize cell nuclei. Sections were analyzed using a Nicon Eclipse 80i microscope.

In the case of immunofluorescence staining the sections were deparaffinized and blocked in 2.5 % BSA. Then sections were incubated with primary antibodies as described above and with secondary donkey anti-rabbit conjugated with Alexa555 or donkey anti-mouse conjugated with Alexa488 (Jackson Immunoresearch). Section were mounted in Vectashield (Vector) and analyzed using Leica TCS SP5 confocal microscope.

### Reverse transcriptase-polymerase chain reaction (RT-PCR)

Total RNA was extracted by homogenization of frozen rat tissues with TRIzol reagent (Invitrogen), followed by centrifugation for 10 min at 12,000 rpm in an Eppendorf centrifuge. Then 2 μg of RNA was reverse transcribed using M-MLV Reverse Transcriptase (Sigma). The sequences of primers for CacyBP/SIP were as follows: forward 5′-AAGACACGGAATTTTGAGGC-3′ and reverse 5′-CACATCACCAGTTCCCATACGG-3′. For GAPDH, sequences of primers were the following: forward 5′- ACCACAGTCCATGCCATCAC-3′ and reverse 5′-TCCACCACCCTGTTGCTGTA-3′. Then the PCR products were separated in 1 % agarose gel containing 0.1 μg/ml ethidium bromide. Pictures of Western blots and agarose gels were taken using the Ingenius BioImaging system (Syngene).

## Results

### Histopathology

**Fig. 1:**
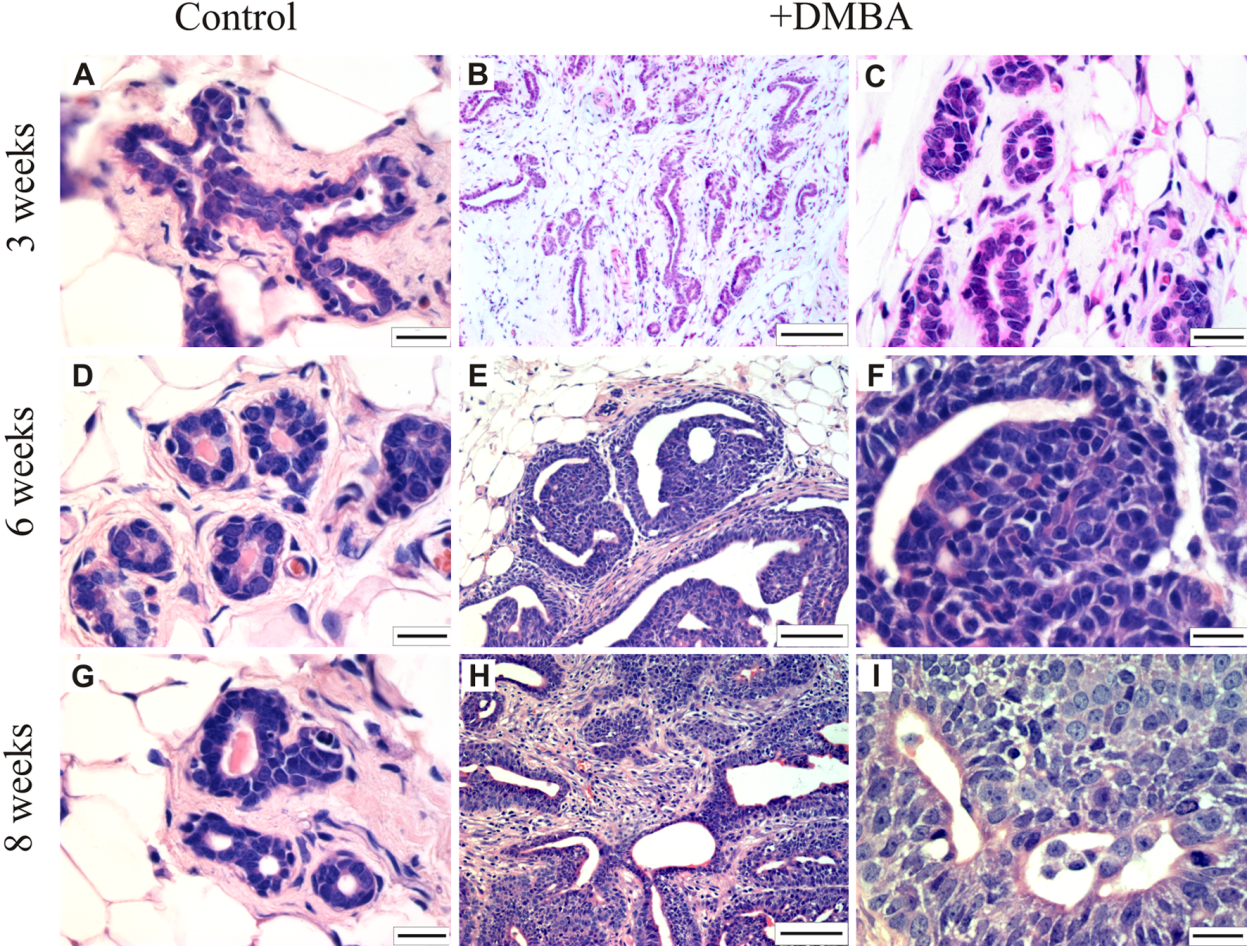
Hematoxylin and eosin staining of rat mammary tissue sections. Normal epithelial ducts are visible in mammary sections taken from rats 3 weeks after PBS (control) (**a**) or DMBA (**b**, **c**) treatment. Normal histology is also seen in mammary sections taken from control rats after 6 and 8 weeks (**d**, **g**), whereas in one mammary sample taken 6 weeks (**e**, **f**) and in all tumors taken 8 weeks after DMBA treatment (**h**, **i**) preinvasive (**e**, **f**) and invasive (**h**, **i**) carcinoma structures were found. Representative images, out of three analyzed for each tissue sample, are shown. The *bar* is 20 μm in the *left* and *right panels* and 100 μm in the *middle panel*

In all mammary sections taken at different time points from the control animals (treated with PBS) as well as in mammary sections taken 3 weeks after treatment with DMBA, normal epithelial histology was observed (Fig. 1a, d, g and b, c, respectively). Initial changes resembling tumor tissue were observed only in one out of two mammary samples taken from one rat 6 weeks after DMBA treatment. As shown in Fig. 1e and f, small, preinvasive carcinoma is detected with the predominance of ductal carcinoma in situ structures. This pathologically changed tissue, obtained from a rat 6 weeks after DMBA treatment, was then used for anti-CacyBP/SIP staining and for Western blotting and RT-PCR analysis. Eight weeks after DMBA treatment both rats developed tumors. Three tumors were found in one rat (1.2, 0.4, and 0.6 cm in diameter) and four in the other (1, 1, 2.2, and 1.2 cm in diameter). All tumors displayed features of ductal invasive carcinoma and diverse morphology. Within some tumors, cribriform, papillous, and pseudopapillous structures were seen (Fig. 1h, i). The histological grade of each tumor was determined as grade 2, based on tubule formation, nuclear size, and mitotic count.

### Distribution and level of CacyBP/SIP at different stages of rat breast cancer development

**Fig. 2:**
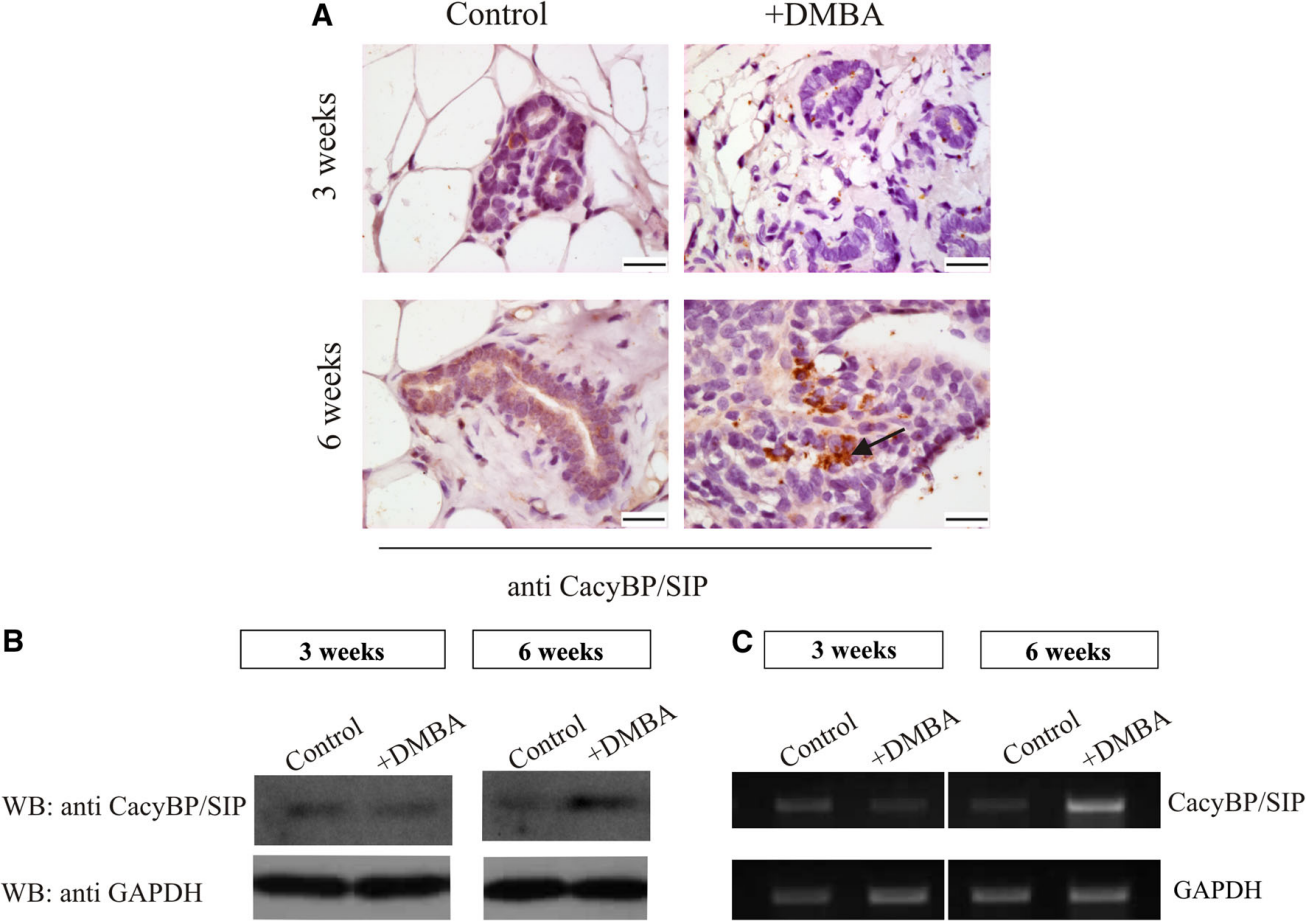
**a** Immunohistochemical staining of CacyBP/SIP in mammary tissues of control and DMBA-treated rats taken 3 and 6 weeks after treatment. Two tissue samples from one control and two DMBA-treated animals were examined and representative imagines are shown. Weak cytoplasmic staining is seen in mammary sections from control or DMBA-treated rats after 3 weeks (*upper panels*) and in a control section taken after 6 weeks (*left*, *lower panel*). In one mammary sample taken from a rat 6 weeks after DMBA treatment the CacyBP/SIP immunoreactivity was more intensive and was seen in the form of cytoplasmic deposits in a small population of cancer cells (*right*, *lower panel*, *arrow*). The *bar* is 20 μm. **b** Western blot showing the level of CacyBP/SIP in mammary tissues of control or DMBA-treated rats taken 3 or 6 weeks after treatment. 40 μg of protein was applied on the SDS gel. Staining with anti-GAPDH antibody shows that each lane contains a similar amount of proteins. Representative immunoblots for samples taken from the control and 3 week DMBA-treated animals are shown, whereas in the case of 6 week DMBA-treated animals an immunoblot representative of the sample showing preinvasive changes is shown. **c** Results of RT-PCR showing the level of mRNA for CacyBP/SIP. The level of mRNA for GAPDH indicates that a similar amount of total RNA was used in the reaction. Representative analyses, out of two performed, of samples taken from the control and 3 week DMBA-treated animals are shown. In the case of 6 week DMBA-treated animals a representative analysis, out of two performed, for the sample showing preinvasive changes is shown

Immunohistochemical staining with anti-CacyBP/SIP antibodies of sections obtained from mammary tissues, either control or 3 weeks after DMBA treatment, revealed the presence of this protein at the vestigial level and was limited only to few granules localized in normal duct epithelial cells and stroma (Fig. 2a). On the contrary, in sections of the mammary sample taken 6 weeks after DMBA treatment, which showed histopathological changes, the CacyBP/SIP immunoreactivity was more prominent, present in a form of deposits in the cytoplasm of cancer cells (Fig. 2a, lower right panel). Similar results, showing an increase in the level of the CacyBP/SIP protein and its mRNA in this sample, were obtained using Western blotting (Fig. 2b) and RT-PCR (Fig. 2c).

**Fig. 3:**
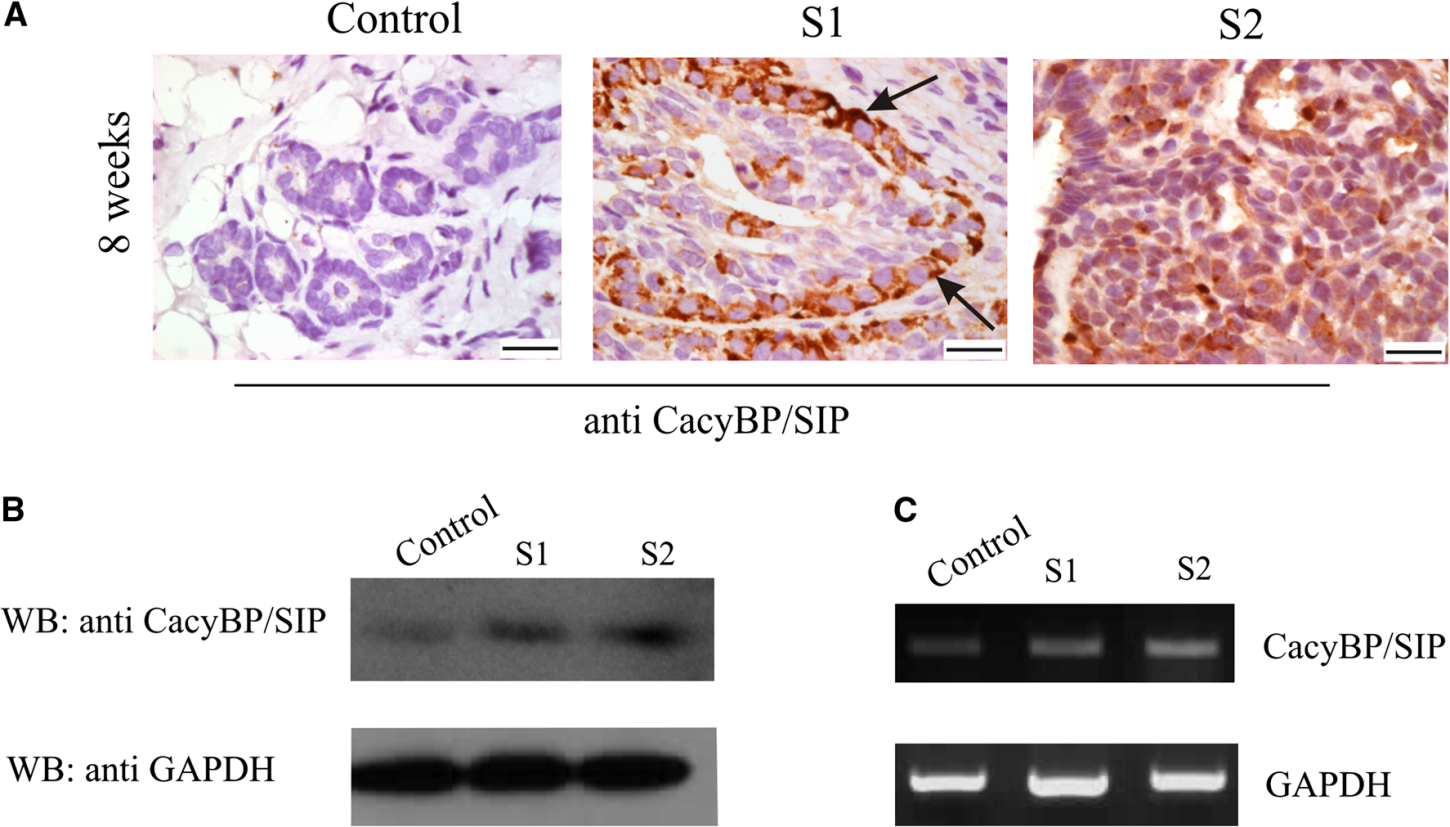
**a** Immunohistochemical staining of CacyBP/SIP in control and tumor sections taken 8 weeks after DMBA treatment. The CacyBP/SIP immunoreactivity in tumor samples *S1* and *S2* (sections prepared from two different tumors taken from two different rats) is present predominantly in the cytoplasm of cancer cells localized at the basal layer of ductal structures (*arrows* in *S1*). Representative images, out of three analyzed for each tissue sample, are shown. *Bar* is 20 μm. **b** Western blot showing the level of CacyBP/SIP in the control mammary tissue and tumor samples (*S1* and *S2*) taken 8 weeks after DMBA treatment. 40 μg of protein was applied on the SDS gel. Staining with anti-GAPDH antibody shows that each lane contains a similar amount of protein. A representative immunoblot is shown. **c** Results of RT-PCR showing the level of mRNA for CacyBP/SIP. The level of mRNA for GAPDH indicates that a similar amount of total RNA was used in the reaction. A representative RT-PCR analysis, out of two performed, for each sample is shown

Immunohistochemical staining with anti-CacyBP/SIP antibodies of sections prepared from mammary tumors taken 8 weeks after DMBA treatment was similar to the immunostaining of sections prepared from the pathologically changed mammary tissue taken 6 weeks after DMBA treatment, but the CacyBP/SIP immunoreactivity was much more intensive. Furthermore, in sections from two different tumors (S1 and S2) taken from two different rats 8 weeks after DMBA treatment, especially high CacyBP/SIP immunoreactivity was seen in cells localized at the basal layer of the ductal structures (Fig. 3a). An increase in the CacyBP/SIP protein and mRNA level was confirmed by Western blotting (Fig. 3b) and RT-PCR (Fig. 3c).

### Correlation between CacyBP/SIP and β-catenin level in breast cancer tissues

**Fig. 4:**
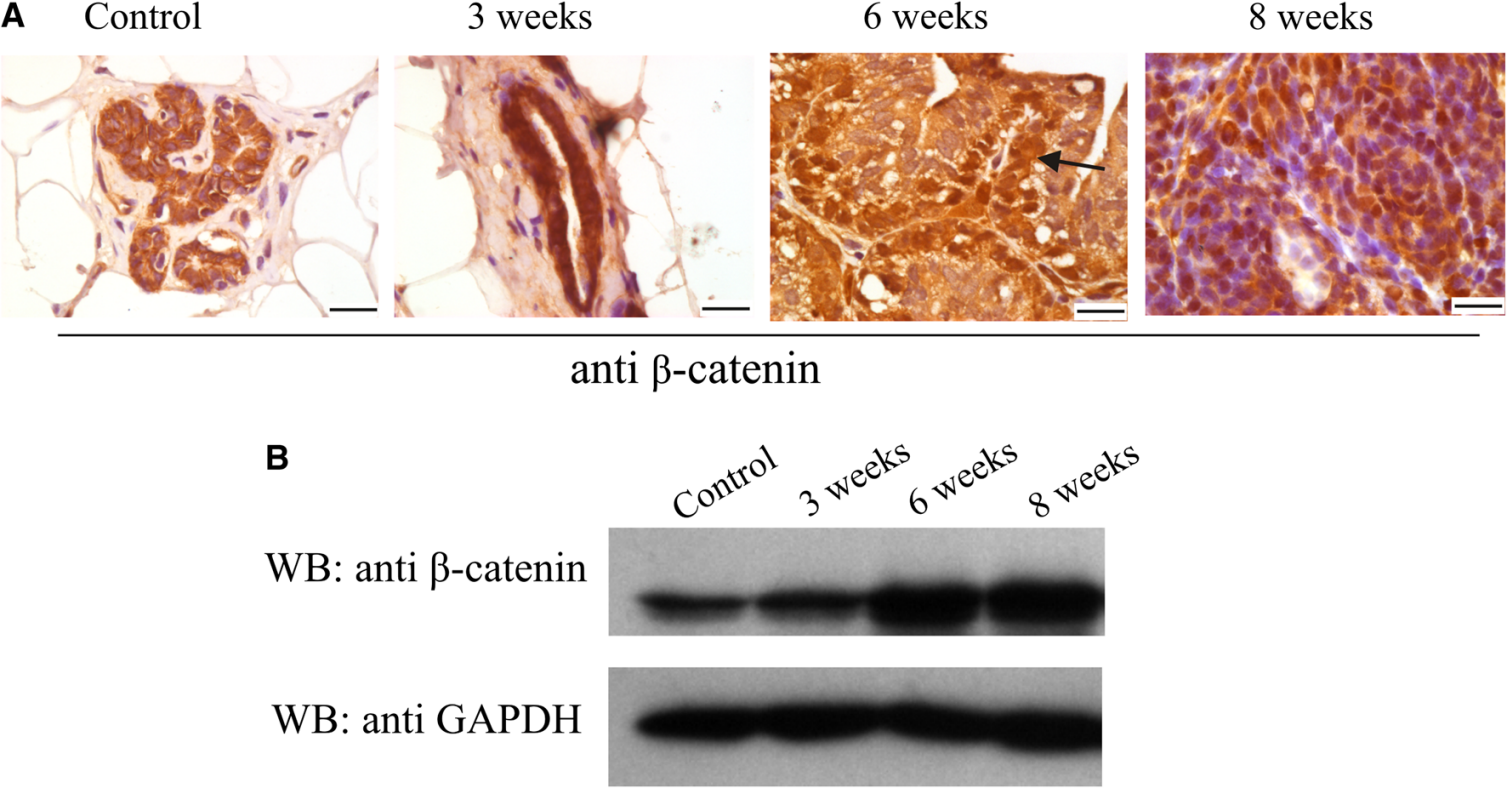
**a** Immunohistochemical staining with anti-β-catenin antibody. In the control section immunoreactivity is present in the cytoplasm and cell membranes of epithelial cells. In sections obtained from mammary samples taken 3, 6, and 8 weeks after DMBA treatment the immunoreactivity is increased in nuclei of cancer cells localized at the basal layer of the ductal, cribriform, and papillary structures (*arrow*). The *bar* is 20 μm. Representative images, out of three analyzed for each tissue sample, are shown. **b** Western blot showing the level of β-catenin in the control mammary tissue and in tumor samples taken 3, 6, and 8 weeks after DMBA treatment. 40 μg of protein was applied on the SDS gel. Staining with anti-GAPDH antibody shows that each lane contains a similar amount of protein. A representative immunoblot, out of three performed for samples shown in Fig. 4a, is presented

In order to check whether the increased level of CacyBP/SIP is correlated with that of an oncogene, β-catenin, we examined the tissue samples for the presence of the latter protein. The immunostaining of β-catenin in control mammary tissue was uniformly distributed among the epithelial cells and was mainly seen in the cytoplasm and in the cell membrane (Fig. 4a). In section prepared from mammary tissues taken 3, 6, and 8 weeks after DMBA treatment, the immunoreactivity is increased in nuclei of cancer cells localized at the basal layer of the ductal, cribriform, and papillary structures with the highest intensity in those taken at 8 weeks. An increase in β-catenin level in mammary tissues obtained from rats treated with DMBA was also confirmed by Western blot analysis (Fig. 4b).

**Fig. 5:**
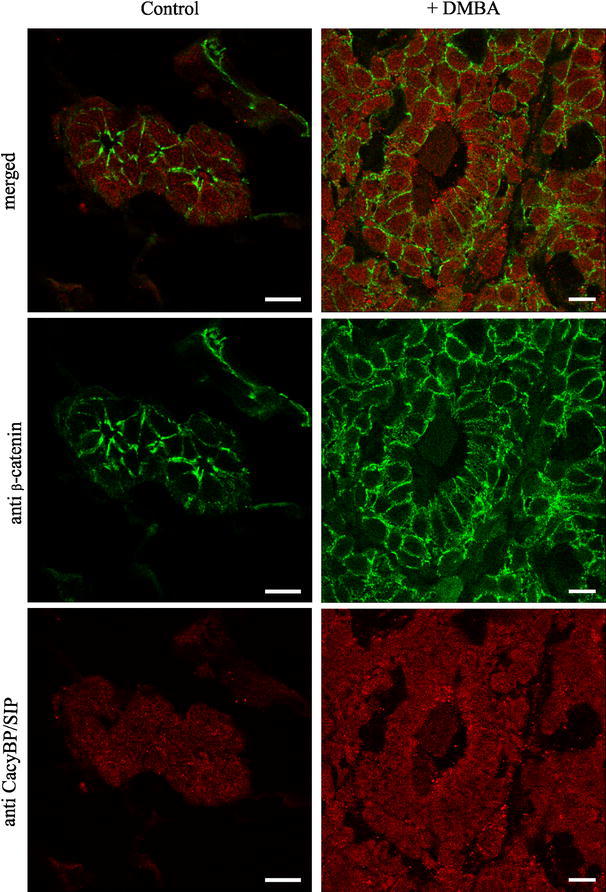
Immunofluorescence staining of β-catenin and CacyBP/SIP in sections of control and DMBA-treated rats. β-Catenin is shown in *green* and CacyBP/SIP is in *red*. The *bar* is 10 μm. Representative images, out of three analyzed for control and S1 sample taken from 8 week DMBA-treated animal, are shown

Although, the level of both proteins, β-catenin and CacyBP/SIP, was increased in cancer tissue, detailed immunofluorescence analysis showed that they had distinct subcellular localization (Fig. 5). β-Catenin was clearly visible in areas of cell-to-cell contact, whereas CacyBP/SIP was localized in the cytoplasm with more intense staining in apical cell areas.

## Discussion

Breast cancer is the most common malignancy among women worldwide and is the most common cause of death for women between 35 and 50 years of age. Women with breast cancer are at risk of developing metastases for their entire lifetime [20]. Despite advances in genetic and biochemical analyses, the precise mechanism of mammary carcinogenesis is unknown. Thus, a better understanding of the mechanisms and signals involved in breast cancer progression could lead to the development of more targeted therapies to inhibit the pathways or some molecules that promote disease progression [21]. One approach is to search for novel molecules/proteins which might regulate and be important in carcinogenesis.

Regarding the protein of our interest, CacyBP/SIP, there are several reports showing its possible involvement in carcinogenesis. In some cases CacyBP/SIP seems to function as a tumor suppressor, i.e., in gastric or renal cell carcinoma [12], and in other cases, such as pancreatic or colon cancer, as an oncogene [9, 13]. In the case of breast cancer there are only two papers concerning this protein and they yield contradictory data. One group indicated that a lower level of CacyBP/SIP was present in the tumor tissue in comparison to the control one and, moreover, reduction in CacyBP/SIP expression was associated with poor prognosis of breast cancer patients [15]. In contrast, Wang and co-workers [16] suggested that CacyBP/SIP was up-regulated in breast cancer tissues. To further explore the problem of CacyBP/SIP expression in breast carcinogenesis, we decided to check CacyBP/SIP expression both at the mRNA and protein level in breast cancer tumors induced by DMBA. This approach allowed us to monitor CacyBP/SIP expression at different stages of breast cancer development and we found, using this model, that CacyBP/SIP is up-regulated during carcinogenesis. The most interesting result is that the expression of CacyBP/SIP, at both mRNA and protein levels, was quite well detectable in the mammary tissue with only minimal pathological changes obtained 6 weeks after DMBA treatment. This observation suggests that CacyBP/SIP plays an important role in breast carcinogenesis at a very early stage of its development. Some earlier studies indicated that CacyBP/SIP might modulate the malignant cell behavior by regulating the level of β-catenin [22]. β-Catenin is a protein which, besides being involved in cell adhesion, may act as an oncogene in different types of tumor [23]. Stabilization and accumulation of β-catenin in the cell, due to activation of the Wnt pathway [24], promotes nuclear translocaton of β-catenin and its binding to TCF/LEF (T cell factor/leukocyte enhancing factor 1) which in consequence activates genes important for cell proliferation such as *cyclin D1*. Notably, this gene is amplified in about 20 % of human breast cancers [25] and the cyclin D1 protein is expressed at increased level in about 50 % of human breast tumors [26].

In this work we found that the level of CacyBP/SIP was increased in rat mammary cancer tissues similarly to the level of β-catenin, although both proteins appeared to have distinct subcellular localization. To uncover the role of CacyBP/SIP in breast cancer and to elucidate the possible interrelationship between these two proteins further studies are needed.

## Abbreviations

DMBA: Dimethylbenz[*a*]anthracene
DTT: Dithiothreitol
GAPDH: Glyceraldehyde 3-phosphate dehydrogenase
PAGE: Polyacrylamide gel electrophoresis
SDS: Sodium dodecyl sulfate
TBS: Tris buffered saline

## References

[1] Filipek A, Wojda U (1996). p30, a novel protein target of mouse calcyclin (S100A6). Biochem J.

[2] Schneider G, Filipek A (2011). S100A6 binding protein and Siah-1 interacting protein (CacyBP/SIP): spotlight on properties and cellular function. Amino Acids.

[3] Kilanczyk E, Filipek S, Jastrzębska B, Filipek A (2009). CacyBP/SIP binds ERK1/2 and affects transcriptional activity of Elk-1. Biochem Biophys Res Commun.

[4] Kilanczyk E, Filipek S, Filipek A (2011). ERK1/2 is dephosphorylated by a novel phosphatase-CacyBP/SIP. Biochem Biophys Res Commun.

[5] Schneider G, Nieznanski K, Kilanczyk E, Bieganowski P, Kuznicki J, Filipek A (2007). CacyBP/SIP interacts with tubulin in neuroblastoma NB2a cells and induces formation of globular tubulin assemblies. Biochim Biophys Acta.

[6] Schneider G, Nieznanski K, Jozwiak J, Slomnicki LP, Redowicz MJ, Filipek A (2010). Tubulin binding protein, CacyBP/SIP, induces actin polymerization and may link actin and tubulin cytoskeletons. Biochim Biophys Acta.

[7] Au KW, Kou CY, Woo AY, Chim SS, Fung KP, Cheng CH (2006). Calcyclin binding protein promotes DNA synthesis and differentiation in rat neonatal cardiomyocytes. J Cell Biochem.

[8] Lou JR, Zhang XX, Zheng J, Ding WQ (2010). Transient metals enhance cytotoxicity of curcumin: potential involvement of the NF-kappaB and mTOR signaling pathways. Anticancer Res.

[9] Chen X, Han G, Ziai H, Hang F, Wang J, Li X (2008). Expression and clinical significance of CacyBP/SIP in pancreatic cancer. Pancreatology.

[10] Zhai H, Shi Y, Jin H, Li Y, Lu Y, Chen X (2008). Expression of calcyclin-binding protein/Siah-1 interacting protein in normal and malignant human tissues: an immunohistochemical survey. J Histochem Cytochem.

[11] Chen X, Mo P, Li X, Zheng P, Zhao L, Xue Z, et al. CacyBP/SIP protein promotes proliferation and G1/S transition of human pancreatic cancer cells. Mol Carcinog. doi:10.1002/mc.20737. 10.1002/mc.20737 21268134

[12] Sun S, Ning X, Liu J, Liu L, Chen Y, Han S (2007). Overexpressed CacyBP/SIP leads to the suppression of growth in renal cell carcinoma. Biochem Biophys Res Commun.

[13] Ghosh D, Yu H, Tan XF, Lim TK, Zubaidah RM, Tan HT (2011). Identification of key players for colorectal cancer metastasis by iTRAQ™ quantitative proteomics profiling of isogenic SW480 and SW620 cell lines. J Proteome Res.

[14] Kilanczyk E, Wasik U, Filipek A. CacyBP/SIP phosphatase activity in neuroblastoma NB2a and colon cancer HCT116 cells. Biochem Cell Biol. 2012;90:558–64. 10.1139/o2012-011 22480271

[15] Nie F, Yu XL, Wang XG, Tang YF, Wang LL, Ma L (2010). Down-regulation of CacyBP is associated with poor prognosis and the effects on COX-2 expression in breast cancer. Int J Oncol.

[16] Wang N, Ma Q, Wang Y, Ma G, Zhai H (2010). CacyBP/SIP expression is involved in the clinical progression of breast cancer. World J Surg.

[17] Escrich E (1987). Validity of the DMBA-induced mammary cancer model for the study of human breast cancer. Int J Biol Markers.

[18] Barros AC, Muranaka EN, Mori LJ (2004). Induction of experimental mammary carcinogenesis in rats with 7,12-dimethylbenz(a)anthracene. Rev Hosp Clin Fac Med Sao Paulo.

[19] Laemmli UK (1970). Cleavage of structural proteins during the assembly of the head of bacteriophage T4. Nature.

[20] Castaño Z, Tracy K, McAllister SS (2011). The tumor macroenvironment and systemic regulation of breast cancer progression. Int J Dev Biol.

[21] Imamura T, Hikita A, Inoue Y (2011). The roles of TGF-b signaling in carcinogenesis and breast cancer metastasis. Breast Cancer.

[22] Ning X, Sun S, Hong L, Liang J, Liu L, Han S (2007). Calcyclin-binding protein inhibits proliferation, tumorigenicity, and invasion of gastric cancer. Mol Cancer Res.

[23] Fuchs SY, Ougolkov AV, Spielgelman VS, Minamoto T (2005). Oncogenic beta-catenin signaling networks in colorectal cancer. Cell Cycle.

[24] Polakis P (2007). The many ways of WNT in cancer. Curr Opin Genet Dev.

[25] Dickson C, Fantl V, Gillett C, Brookes S, Bartek J, Smith R (1995). Amplification of chromosome band 11q13 and a role for cyclin D1 in human breast cancer. Cancer Lett.

[26] Rajabi H, Ahmad R, Jin C, Kosugi M, Alam M, Joshi MD (2012). MUC1-C oncoprotein induces TCF7L2 activation and promotes cyclin D1 expression in human breast cancer cells. J Biol Chem.

